# Stress-Induced Nuclear RNA Degradation Pathways Regulate Yeast Bromodomain Factor 2 to Promote Cell Survival

**DOI:** 10.1371/journal.pgen.1004661

**Published:** 2014-09-18

**Authors:** Kevin Roy, Guillaume Chanfreau

**Affiliations:** Department of Chemistry and Biochemistry and the Molecular Biology Institute, University of California, Los Angeles, Los Angeles, California, United States of America; Case Western Reserve University, United States of America

## Abstract

Bromodomain proteins are key regulators of gene expression. How the levels of these factors are regulated in specific environmental conditions is unknown. Previous work has established that expression of yeast Bromodomain factor 2 (*BDF2*) is limited by spliceosome-mediated decay (SMD). Here we show that *BDF2* is subject to an additional layer of post-transcriptional control through RNase III-mediated decay (RMD). We found that the yeast RNase III Rnt1p cleaves a stem-loop structure within the *BDF2* mRNA to down-regulate its expression. However, these two nuclear RNA degradation pathways play distinct roles in the regulation of *BDF2* expression, as we show that the RMD and SMD pathways of the *BDF2* mRNA are differentially activated or repressed in specific environmental conditions. RMD is hyper-activated by salt stress and repressed by hydroxyurea-induced DNA damage while SMD is inactivated by salt stress and predominates during DNA damage. Mutations of *cis*-acting signals that control SMD and RMD rescue numerous growth defects of cells lacking Bdf1p, and show that SMD plays an important role in the DNA damage response. These results demonstrate that specific environmental conditions modulate nuclear RNA degradation pathways to control *BDF2* expression and Bdf2p-mediated gene regulation. Moreover, these results show that precise dosage of Bromodomain factors is essential for cell survival in specific environmental conditions, emphasizing their importance for controlling chromatin structure and gene expression in response to environmental stress.

## Author Summary

Cells adapt to changes in the environment through modulating gene expression at both the RNA and protein levels. RNA degradation plays a central role in this adaption response, by controlling the stability of specific mRNAs to optimize protein production in different conditions. In this study, we show that the gene encoding Bromodomain Factor 2 (*BDF2*) is tightly regulated according to environmental conditions by two distinct RNA degradation mechanisms. We show that these RNA degradation pathways are critical for cell growth in specific conditions. Our study suggests that environmental modulation of nuclear RNA degradation pathways is a previously unappreciated aspect of gene expression control.

## Introduction

DNA in eukaryotes is wrapped around histone octamers to form nucleosomes [1]. The tails of the histone proteins are subject to a diverse set of chemical modifications, including acetylation, phosphorylation, methylation, and ubiquitination, impacting the majority of DNA-based processes, including transcription, heterochromatin formation, DNA replication, and DNA recombination and repair [2], [3]. Non-histone proteins recognize specific tail modifications to mediate the downstream effects [4]. Histone lysine acetylation, one of the best-studied modifications, has important roles in transcription activation, DNA repair and heterochromatin formation [5]. Histone acetylation can increase accessibility of DNA by weakening the interaction between the positively charged histone tail and the nucleosomal DNA [6]. Histone acetylation can also recruit proteins containing bromodomains, which are evolutionarily conserved motifs that recognize acetyl-lysines and play an important role in anchoring chromatin-associated complexes to the nucleosome [7].

*S. cerevisiae* bromodomain factors 1 and 2 (Bdf1p and Bdf2p) localize throughout the genome at loci enriched for acetylated histones 3 and 4 [5]; [8], where they function in various aspects of transcription initiation and chromatin remodeling [9], as well as protection of euchromatin against heterochromatin spreading [10]. While the *bdf1* ***Δ*** *bdf2* ***Δ*** double deletion is lethal, both single deletion mutants are viable, indicating that there is at least partial functional redundancy between the two paralogs [11]. In wild-type cells, Bdf1p is present at nearly 3-fold the levels of Bdf2p [12], and each occupies distinct genomic locations [8]. Cells lacking *BDF1* (*bdf1* ***Δ***) show an upregulation of Bdf2 protein (Bdf2p) levels [13] and a redistribution of Bdf2p to the acetylated histones at genomic loci normally bound by Bdf1p [8]. The deletion of *BDF2* affects expression of less than 0.05% of the transcriptome in normal conditions, while the deletion of *BDF1* results in a greater than 2-fold change in the levels of ∼15% of all expressed transcripts [10].

In addition to activating TFIID-dependent transcription [11], Bdf1p is part of the SWR1-C chromatin-remodeling complex responsible for replacing histone H2A with the variant H2AZ [14]. The NuA4 histone acetyltransferase acts upstream, depositing an acetyl group on the histone H4, resulting in the recruitment of Bdf1p and SWR1-C [15]. Recent work has revealed that mutations in NuA4 subunits, Bdf1p, SWR1-C, or H2AZ render cells hypersensitive to DNA damage agents, suggesting that histone acetylation-mediated H2AZ deposition plays crucial roles in the DNA damage response [16]; [17]. Furthermore, cells lacking *BDF1* display a growth defect in normal conditions and hypersensitivity to a wide range of stress conditions [10], [16], [18]. While cells lacking *BDF2* exhibit no growth defect in normal conditions, they display decreased resistance to the DNA damage agents camptothecin and bleomycin [19]. Additionally, recent studies in fission yeast have demonstrated an important role for Bdf2p in the S-phase stress response and the establishment of heterochromatin boundaries [17], [20].

Despite detailed biochemical and functional analysis of the Bdf1p and Bdf2p proteins, little is known about how these factors are regulated in specific environmental conditions. A recent study demonstrated a novel role for the spliceosome in degrading the *BDF2* mRNA, through coupling the splicing of an intron encoded within the *BDF2* ORF to decay by nuclear exonucleases [13]. Spliceosome-mediated decay (SMD) of *BDF2* mRNA was found to be dependent on Bdf1p-mediated recruitment of the spliceosome to the *BDF2* locus, and its proposed biological function was to maintain the homeostasis of Bdf2p levels in normal growth conditions. Here we demonstrate that *BDF2* expression is subject to extensive control by two distinct nuclear RNA degradation pathways, and that each pathway is regulated in different stress conditions to maintain cellular fitness. These results also establish that tight control of the levels of Bromodomain factors is important for cell survival in specific environmental conditions, underscoring their importance for regulating gene expression during stress.

## Results

### The *S.cerevisiae* RNase III Rnt1p cleaves the *BDF2* mRNA *in vivo* and *in vitro*

**Figure 1:**
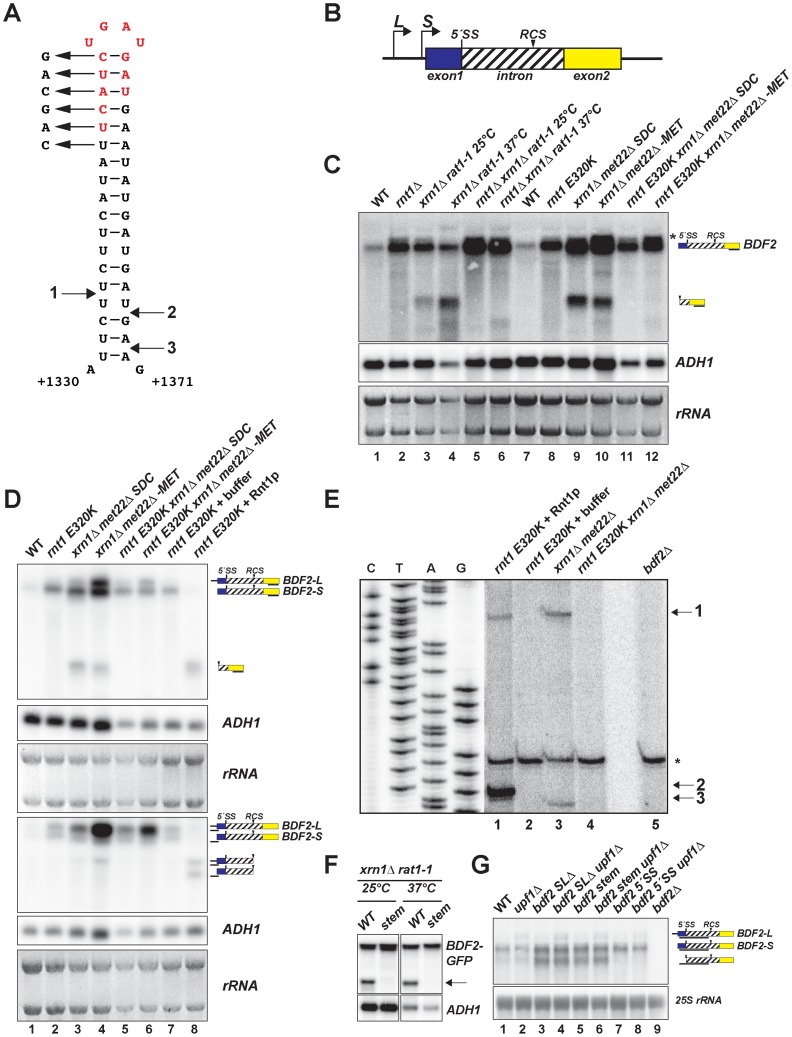
*BDF2* mRNA contains a canonical Rnt1p target stem-loop within its intron and is cleaved by Rnt1p *in vivo* and *in vitro*. **A**. The predicted RNA secondary structure for the *BDF2* transcript is shown for positions +1330 to +1371 of the open reading frame (chr IV: 332354–332395). **B**. Two different transcription start sites give rise to short and long forms of *BDF2* (*-S* and *-L*). The 5′-splice site (5′-SS) and the intronic RNase III cleavage site (RCS) positions are shown relative to exon 1 (blue box) and exon 2 (yellow box). The *BDF2* intron (chr IV: 331189-332695) encodes amino acids 56–557 of the *BDF2* open reading frame, and is depicted as a hashed box to distinguish it from the typical representation of non-coding introns. **C**. Northern blot detecting the full-length *BDF2* transcript and the Rnt1p degradation product with a riboprobe targeting *BDF2* exon2. The nuclear 5′ to 3′ exonuclease Rat1p was inactivated by shifting the thermo-sensitive *rat1-1* strain from 25°C to the non-permissive temperature (37°C) for 2.5 hours (lanes 3–6), or by shifting the *met22Δ* strain from synthetic-dextrose complete (SDC) media to medium lacking methionine (-MET) for 12 hours (lanes 9–12). *ADH1* and the ethidium bromide-stained rRNAs of the gel prior to transfer are shown as loading controls. **D**. Lanes 1–6 are from (C). RNA from the *rnt1 E320K* catalytic mutant was incubated with the presence of buffer only (lane 7) or with recombinant Rnt1p (lane 8). The same samples were loaded in duplicate series and probed for either exon2 (top panel) or the 5′- UTR region (bottom panel). The black line beneath each *BDF2* RNA species indicates the relative position of the probe. **E**. Primer extension mapping of Rnt1p cleavage sites on *BDF2* mRNA *in vitro* and *in vivo*. A primer was designed to hybridize to the *BDF2* transcript downstream of the Rnt1p cleavage site, at position +1436 to +1457 in the open reading frame (chr IV: 332460-332481). The sequencing ladder and lanes 1–5 represent the alignment from two different exposures of the same gel. RNA from the *rnt1 E320K xrn1* ***Δ*** *met22Δ* strain was incubated in the presence of recombinant Rnt1p (lane 1), or buffer only (lane 2) to demonstrate *in vitro* cleavage sites. Lanes 3 and 4 consist of the *xrn1* ***Δ*** *met22Δ* and *rnt1 E320K xrn1* ***Δ*** *met22Δ* strains shifted to –MET to demonstrate *in vivo* Rnt1p cleavage sites. Lane 5 consists of primer extension on RNA from the *bdf2Δ* strain, demonstrating that the asterisked band present in all lanes is a non-specific primer extension product. The Rnt1p cleavage sites are numbered 1 (*in vivo and in vitro*), 2 (*in vitro*), and 3 (*in vivo*) and are mapped onto the stem-loop in (A). **F**. The *BDF2* ORF was cloned into the pUG23 vector with a C-terminal GFP fusion (depicted as a black box) and transformed into the *xrn1* ***Δ*** *rat1-1* background. The top six base pairs of the Rnt1p target stem-loop were disrupted using synonymous base substitutions as shown in (A). The strains were shifted to the non-permissive temperature as in (C). The *BDF2-GFP* fusion transcript was detected with a probe to the *GFP* ORF. The arrow points to the 3′ Rnt1p cleavage product. **G**. Northern blot analysis of WT *BDF2*, 5′-SS mutant (*bdf2 5′-SS*) and Rnt1 stem-loop mutants (*bdf2 SL* ***Δ*** and *bdf2 stem mut*) in the wild-type and *upf1* ***Δ*** backgrounds. An exon1-intron riboprobe detects both *-L and -S* forms of *BDF2*, as well the intron-exon2 cleavage product of spliceosome-mediated decay.

Recent studies have revealed the presence of cryptic splicing signals within *S.cerevisiae* transcripts [13], [21], [22]. In contrast to the canonical role of the spliceosome in promoting gene expression, the usage of these splice sites by the spliceosome promotes degradation of these transcripts [13]. We previously showed that the yeast orthologue of RNase III (Rnt1p) cleaves stem-loop structures within introns of various transcripts to regulate the levels of the spliced transcripts [23]. To test whether these recently identified cryptic introns contain cleavage signals for Rnt1p, we performed a computational RNA secondary structure screen for these intron-containing transcripts using m-Fold [24]. We analyzed the predicted secondary structures for canonical Rnt1p cleavage signals, which consist of double-stranded RNA (dsRNA) hairpin structures capped by NGNN or AAGU tetraloops [25], [26]. This analysis revealed the presence of a canonical Rnt1p stem-loop within the open reading frame (ORF) of the yeast gene encoding bromodomain factor 2 (*BDF2*) (Fig. 1A). This 40 base-long stem-loop is situated within the ORF-embedded intron of the *BDF2* mRNA, 1165 nucleotides downstream of the 5′-SS and 298 nucleotides upstream of the annotated 3′-SS ([13], Fig. 1B). The existence of this stem-loop structure was confirmed *in vivo* in a transcriptome-wide study of RNA secondary structure that employed dimethyl sulfate (DMS) modification of single-stranded RNA coupled to deep sequencing [27]. Consistent with its potential degradation by Rnt1p, microarray analysis showed that the *BDF2* transcript is upregulated 2-fold in cells lacking Rnt1p (*rnt1* ***Δ***; [28]). Furthermore, recent studies have found that *BDF2* mRNA exhibits a high transcription rate in conjunction with a short half-life that is similar to the histone mRNAs, suggesting that RNA degradation may play a major role in its regulation ([29], see Discussion). These initial observations suggested that *BDF2* expression is highly regulated at the level of RNA degradation and raised the possibility that Rnt1p may directly control *BDF2* expression.

To investigate if the upregulation of *BDF2* mRNA in *rnt1* ***Δ*** is specifically due to the loss of Rnt1p catalytic activity, we compared *BDF2* transcripts levels in wild-type (WT) and *rnt1* ***Δ*** strains, and in a strain expressing the catalytically inactive *rnt1 E320K* mutant (Fig. 1C, lanes 1–2, 7–8). This analysis revealed a substantial increase of *BDF2* mRNA upon the loss of Rnt1p catalytic function. To formally demonstrate cleavage of *BDF2* by Rnt1p *in vivo*, we analyzed *BDF2* species in strains carrying mutations in various exonucleases in order to stabilize the 3′ product of Rnt1p cleavage, which is normally subject to decay by 5′-3′ exonucleases due to the presence of an unprotected 5′-end with an exposed monophosphate. The *xrn1* ***Δ*** background was used to inactivate the major pathway of cytoplasmic 5′ to 3′ degradation. To inactivate the nuclear 5′ to 3′ exonuclease Rat1p, we used the temperature-sensitive *rat1-1* mutant. In a parallel approach, we deleted the *MET22* gene in the *xrn1* ***Δ*** background. Upon a shift to medium lacking methionine, this strain accumulates 3′-phosphoadenosine-5′-phosphate (pAp), a metabolite that inhibits cellular 5′ to 3′ exonuclease activities [30]. We utilized a probe hybridizing to the 3′UTR of *BDF2* mRNA and found that both methods of inactivating the 5′ to 3′ exonucleases revealed a substantial accumulation of a species migrating faster than the full-length *BDF2* mRNA (Fig. 1C, lanes 3,4,9,10). This species was no longer detected upon disruption of *RNT1* in these strains (Fig. 1C, lanes 5,6,11,12) suggesting that it is the downstream (3′) product of Rnt1p cleavage.

We detected a slower migrating form of *BDF2* mRNA in samples from *rnt1* mutant backgrounds (Fig1C, asterisk). Previous studies had identified two different transcription start sites for *BDF2* corresponding to two distinct nucleosome free regions (NFRs) [31]–[33]. To confirm that two different forms of *BDF2* mRNA differing by their 5′-UTR were present, we performed prolonged electrophoresis in high percentage gels (2% agarose). This enabled the detection of two closely migrating forms of the *BDF2* mRNA (denoted *BDF2-L* and *BDF2*-S), the longer of which was up-regulated in the *met22* ***Δ*** background after a shift to medium lacking methionine (Fig. 1D, upper panel, lanes 4,6). This analysis confirmed that *BDF2-S* is the predominant form in wild-type cells under normal conditions. We subsequently used probes targeted to the 5′UTR of the transcript arising from the upstream transcription start site (discussed below), to demonstrate that *BDF2-L* is indeed a 5′-extended form of *BDF2-S*.

We next tested whether Rnt1p could directly cleave the *BDF2* mRNA *in vitro* by incubating recombinant Rnt1p enzyme with total RNAs extracted from a strain expressing the catalytic mutant *rnt1 E320K* (Fig. 1D, lanes 7–8). The 3′-UTR probe detected a single band from the *in vitro* cleavage reaction, and this band co-migrated with the band stabilized *in vivo* in the *xrn1* ***Δ*** background (Fig. 1D, upper panel, lanes 3,4, and 8). Next, we utilized a 5′UTR probe to detect the upstream product of Rnt1p cleavage. This probe was designed to bind predominantly to *BDF2-L*, and with only a short stretch of complementarity to the 5′-end of *BDF2-S*. This resulted in an enhanced signal for *BDF2-L* relative to *BDF2-S*, consistent with *BDF2-L* having a 5′-extension. Furthermore, *in vitro* cleavage of total RNAs by Rnt1p gave rise to two different 5′-products, the longer of which was also observed as an *in vivo* cleavage product in the *xrn1* ***Δ*** *met22* ***Δ*** background (Fig. 1D, lower panel, lane 4). This was surprising as the 5′ cleavage product generated by Rnt1p exhibits an exposed 3′-OH, lacks a stabilizing poly(A) tail, and is subject to degradation by 3′ to 5′ exonucleases [23], [34]. The stabilization of this species upon depletion of 5′ to 3′ exonuclease activity may be an indirect consequence of 3′ to 5′ exonucleases being saturated by RNA substrates in these conditions. We tested the ability of Rnt1p to cleave the mRNA encoding the *BDF2* paralog (*BDF1*), and other targets of spliceosome-mediated decay [13] identified in our computational screen, but found no evidence for *in vivo* or *in vitro* cleavage of these transcripts (Fig. S1).

To precisely identify the Rnt1p cleavage site on *BDF2* mRNA, we performed primer extension analysis using a primer hybridizing 70 nucleotides downstream of the stem-loop structure. We detected two primer extension stops from *in vitro* cleavage with recombinant Rnt1p (Fig. 1E), corresponding to 14 and 15 bases below the tetraloop for the 5′ and 3′ cleavage sites, respectively (Fig. 1A and 1E, arrows 1 and 2). Primer extension analysis using RNAs extracted from the 5′ to 3′ exonuclease mutants detected the same site *in vivo* for the 5′ cleavage, and a 3′ cleavage site 17 nucleotides below the tetraloop (Fig. 1E lane 3 and 1A, arrow 3). The distances from the cleavage site to the tetraloop are consistent with the known enzymatic properties of the enzyme *in vitro* and *in vivo* ([25] [35]). Importantly, these cleavage sites were no longer detected when Rnt1p was inactivated in the 5′ to 3′ exonuclease mutants (Fig. 1E, lane 4), showing that they are dependent on Rnt1p activity.

To confirm that this stem loop structure is required for Rnt1p cleavage, we constructed a plasmid-borne version of *BDF2* fused at the C-terminus to GFP, and disrupted the top six base pairs with synonymous mutations (Fig. 1A). We transformed plasmids expressing either the wild-type *BDF2* mRNA or the stem mutant into the *xrn1* ***Δ*** *rat1-1* background and tested for the presence of an Rnt1p cleavage product from these constructs. We utilized a GFP probe to avoid hybridization with endogenous *BDF2* mRNA, and detected the Rnt1p degradation intermediate from the WT *BDF2* construct, which was not observed after disruption of the Rnt1p stem loop (stem mutant, Fig. 1F). These results confirmed that Rnt1p degrades *BDF2* mRNA *in vivo* by recognition of a canonical Rnt1p-target stem loop.

### Mutation of the Rnt1p-target stem loop upregulates *BDF2-S* and stabilizes the intron-exon 2 degradation intermediate of spliceosome-mediated decay (SMD)

A previous study demonstrated that cytoplasmic nonsense-mediated decay (NMD) and nuclear degradation systems can have partially redundant roles in the degradation of various unspliced pre-mRNAs, such that only upon inactivation of both systems does substantial accumulation of these species occur [36]. We found a slight increase in the *BDF2-L* form upon deletion of the gene coding for the NMD helicase Upf1p (*upf1Δ*; Fig. 1G), consistent with the presence of multiple upstream ORFs in the extended 5′ UTR that would elicit recognition of a premature-termination codon and NMD in accordance with the *faux*-3′ UTR model [37]. To test whether NMD cooperates with SMD or Rnt1p in the regulation of *BDF2* mRNA, we mutated the *BDF2* 5′-splice site (5′-SS) or the Rnt1p-target stem loop at the *BDF2* chromosomal copy in otherwise wild-type or *upf1* ***Δ*** backgrounds (Fig. 1G). During the course of this study, RT-PCR analysis of full-length *BDF2* mRNA and its spliced products generated by SMD revealed that mutating the 5′-side of the top six base pairs of the Rnt1p-target stem loop from UUCAUC to CAGCAG inadvertently created a new 3′ splice site, due to the introduction of two YAG sequences proximal to a polypyrimidine tract, (UUC)_3_ (Fig. S2). As an alternative approach to inactivating Rnt1p cleavage *in cis*, we deleted twelve nucleotides at the top of the stem-loop structure, resulting in the in-frame removal of four codons (UCA-UCU-GAU-GAU) encoding an SSDD amino acid sequence (red letters, Fig. 1A). Both mutations of the Rnt1p stem loop resulted in slightly increased *BDF2-S* mRNA levels (Fig. 1F). These stem-loop mutations did not noticeably affect *BDF2-L* levels in the *upf1* ***Δ*** background, suggesting that NMD is the primary degradation system regulating *BDF2-L*. Strikingly, we observed a band migrating faster than *BDF2-S* in the stem-loop mutants. This band was also detectable at low levels in WT cells, but not in the 5′-SS mutant. This band matches the size expected for the linearized intron–exon2 product of SMD, which would be generated by debranching of the lariat intermediate generated by the first splicing step of the *BDF2* mRNA [13], [21]. To definitively demonstrate the identity of this band as the intron-exon2 species, we performed a probe-walking experiment with probes hybridizing to exon1, the intron, and exon2 (Fig. S3). Only the intronic and exon2 probes detected this band, while all three probes detected the full-length *BDF2* mRNA (Fig. S3). It is remarkable that the intron-exon2 species is readily detectable in the absence of exonuclease mutations, as it contains an exposed 5′-phosphate and should be subject to rapid 5′ to 3′ decay. The substantial accumulation of this species in stem-loop mutants suggests that a large fraction of *BDF2* mRNA is subject to SMD in the absence of Rnt1p cleavage.

### Rrp6 and the nuclear retention factors Mlp1/2 regulate *BDF2-S*

We next tested whether the nuclear exosome co-factor Rrp6p and NMD have functional overlap in regulating the *BDF2* mRNA. We found that while both repress *BDF2-L*, there is no additive increase upon inactivation of both degradation systems (Fig. S4A, right panel). The loss of Rrp6p also resulted in increased *BDF2*-S levels, as well as in stabilization of the SMD and Rnt1p cleavage products (Fig. S4A, left panel). The Mlp proteins have previously been implicated in the nuclear retention of unspliced mRNAs [38]. To test the hypothesis that these proteins mediate the nuclear retention of the unspliced-like full-length *BDF2* mRNA, we inactivated Mlp1p and Mlp2p, both of which function to prevent the export of intron-containing mRNA from the nucleus in a manner that depends on 5′-splice site recognition [38] [36]. The *mlp1* ***Δ*** *mlp2* ***Δ*** strain exhibited an upregulation of *BDF2-S*, suggesting that the leakage of *BDF2-S* out of the nucleus results in diminished nuclear decay (Fig. S4B). Mlp1/2 had no effect on the NMD-sensitive *BDF2-L* transcript, consistent with more efficient export and cytoplasmic degradation of this species. In the *rrp6* ***Δ*** background, the inactivation of Mlp1/2 factors did not affect the levels of full-length *BDF2* mRNA or its degradation intermediates.

### Inactivation of Rnt1p-mediated decay (RMD) of *BDF2* rescues the salt sensitivity of the *bdf1Δ* mutant and its inability to grow on non-fermentable carbon

**Figure 2:**
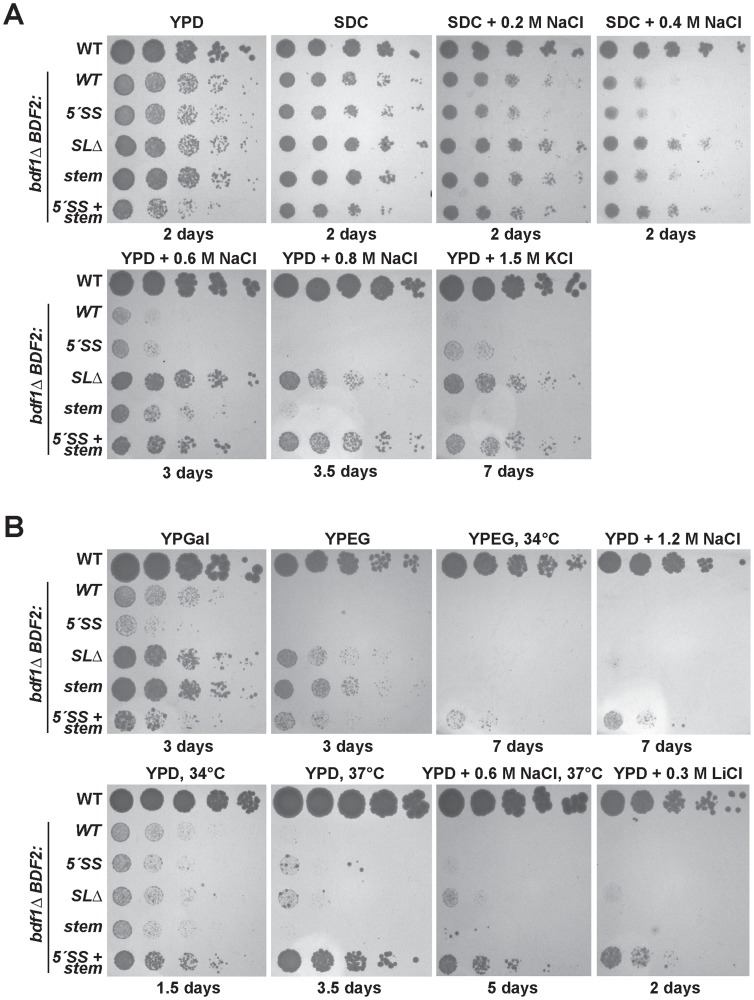
Mutation of the Rnt1p target stem-loop and 5′-splice site suppresses *bdf1Δ* growth defects in various environmental conditions. The top row of spot dilutions in each panel is the wild-type (WT) parent strain. The rest are from the *bdf1* ***Δ*** background with either *WT BDF2*, mutations in the 5′-splice site (*5′-SS*), deletion of the Rnt1p stem-loop (*SL* ***Δ***), mutation of the Rnt1p stem-loop (*stem*), or both (*5′-SS+stem*). Unless indicated otherwise, growth was conducted at 30°C for the indicated number of days. **A**. Inactivating Rnt1p cleavage rescues *bdf1* ***Δ*** salt sensitivity. The differences between the *BDF2* stem mutation (*stem*) and the deletion of the stem (*SL* ***Δ***) are due to an additional *3′-SS* introduced by the stem mutation. **B**. Inactivating Rnt1p cleavage rescues the inability of *bdf1* ***Δ*** to grow on non-fermentable carbon (YPEG) and improves growth on galactose (YPGal). Simultaneous inactivation of both Rnt1p- and spliceosome-mediated decay rescues growth at elevated temperatures and in lithium stress.

In non-fermentable carbon sources or hyperosmotic stress conditions, *BDF2* levels are limiting for growth in *bdf1* ***Δ*** cells, as overexpression of *BDF2* mRNA suppresses *bdf1* ***Δ*** growth defects in a dose-dependent manner [18]. The presence of two distinct nuclear degradation systems acting on the *BDF2* mRNA prompted us to investigate the physiological importance of these degradation pathways in these environmental conditions. A previous study found that blocking SMD of *BDF2* mRNA by mutating the 5′-SS improved the growth of *bdf1* ***Δ*** in the presence of 0.6 M NaCl [13]. To compare the consequences of SMD inactivation and RMD inactivation on *bdf1* ***Δ*** growth phenotypes, we deleted *BDF1* in the *BDF2* 5′-SS mutant (*bdf2 5′SS mut*), the stem-loop mutants (*bdf2 SL* ***Δ*** and *bdf2 stem*), and the double mutant (*bdf2 5′SS mut*+*stem*). In synthetic minimal medium (SDC), but not rich medium (YPD), we found that inactivation of RMD by the stem-loop deletion (*bdf2 SL* ***Δ***) resulted in a modest growth enhancement over wild-type *BDF2* (Fig. 2A, top panel). By contrast, the 5′-SS mutant grew at the same rate as the WT in both of these conditions, consistent with previous observations using plasmid-borne *BDF2* [13].

At 0.2 M and 0.4 M NaCl, the inactivation of RMD resulted in a slight rescue of the *bdf1* ***Δ*** growth defect, with SMD inactivation showing no effect (Fig. 2A, top panel). The simultaneous inactivation of SMD and RMD showed no additional effect over the stem loop deletion strain in these conditions. Surprisingly, at 0.6 M NaCl, the inactivation of RMD resulted in a substantial growth rescue (Fig. 2A, middle panel). The stem loop mutation (which incidentally introduced an additional 3′-SS) resulted in a reduced rescue compared to the stem loop deletion. Inactivating the 5′-SS in the stem mutant resulted in a similar rescue to that of the stem loop deletion, suggesting that the presence of the new 3′-SS site enhanced the SMD of *BDF2* mRNA in these conditions. Importantly, the growth rescue observed upon the deletion of the stem-loop confirmed that the removal of the SSDD amino acid sequence from the Bdf2p protein did not perturb protein function, validating the use of this mutant to assay the phenotypic consequences of increased *BDF2* expression due to RMD inactivation.

We next tested different carbon sources, and found that inactivation of RMD, but not SMD, restored the growth of *bdf1* ***Δ*** cells on the non-fermentable carbon sources ethanol and glycerol (Fig. 2B, top panel). Furthermore, inactivation of RMD conferred a substantial growth rescue in galactose, for which optimal growth requires simultaneous respiration and fermentation [39]. By contrast, disabling the 5′-SS failed to rescue growth in galactose, and slightly exacerbated the growth defect of *bdf1* ***Δ***. It is possible that regulation by SMD occurs at a different phase of the cell cycle than RMD, and that inactivating SMD results in toxic levels of Bdf2p at a particular stage in the presence of galactose. On the other hand, RMD inactivation might increase levels of Bdf2p at a stage where more Bdf2p is beneficial. Overall, these results suggest that the underlying basis for *bdf1* ***Δ*** growth defects in hyper-osmotic stress and respiratory conditions is the degradation of *BDF2* mRNA by RNase III.

### Simultaneous inactivation of the RMD and SMD pathways of *BDF2* suppresses the hypersensitivity of the *bdf1Δ* mutant to elevated temperatures, high salt and lithium stress

While the inactivation of *BDF2* RMD rescued *bdf1Δ* growth defects in moderate levels of salt stress, we found that only the simultaneous inactivation of RMD and SMD allowed the *bdf1Δ* strain to grow at elevated temperatures in non-fermentable carbon (34°C and 37°C), or in high salt stress (1.2M NaCl) (Fig. 2B). We also tested lithium chloride stress, as it confers not only osmotic stress but also lithium ion toxicity. Strains lacking Bdf1p are sensitive to 0.1 M lithium chloride and hyper-accumulate lithium ions [40], [41]. The simultaneous inactivation of RMD and SMD of *BDF2* mRNA enabled growth of *bdf1Δ* at 0.3 M lithium chloride (Fig. 2B, bottom right). These results suggest that in these conditions, both SMD and RMD play significant roles in limiting *BDF2* expression, as inactivation of either degradation pathway alone is insufficient to enhance the growth of *bdf1Δ* cells. Furthermore, these results suggested that either *bdf1* ***Δ*** cells require more *BDF2* mRNA to survive in stress conditions than is required for growth in normal conditions, or that these stress conditions enhance the activity of the nuclear degradation pathways of *BDF2* mRNA, resulting in reduced levels of *BDF2* mRNA relative to normal growth conditions. As cells lacking *BDF1* are strictly dependent on *BDF2* for survival, we hypothesized that stress-induced degradation of *BDF2* mRNA might be responsible for the phenotypes detected in the *bdf1* ***Δ*** strain.

### RMD limits *BDF2* expression in osmotic stress

**Figure 3:**
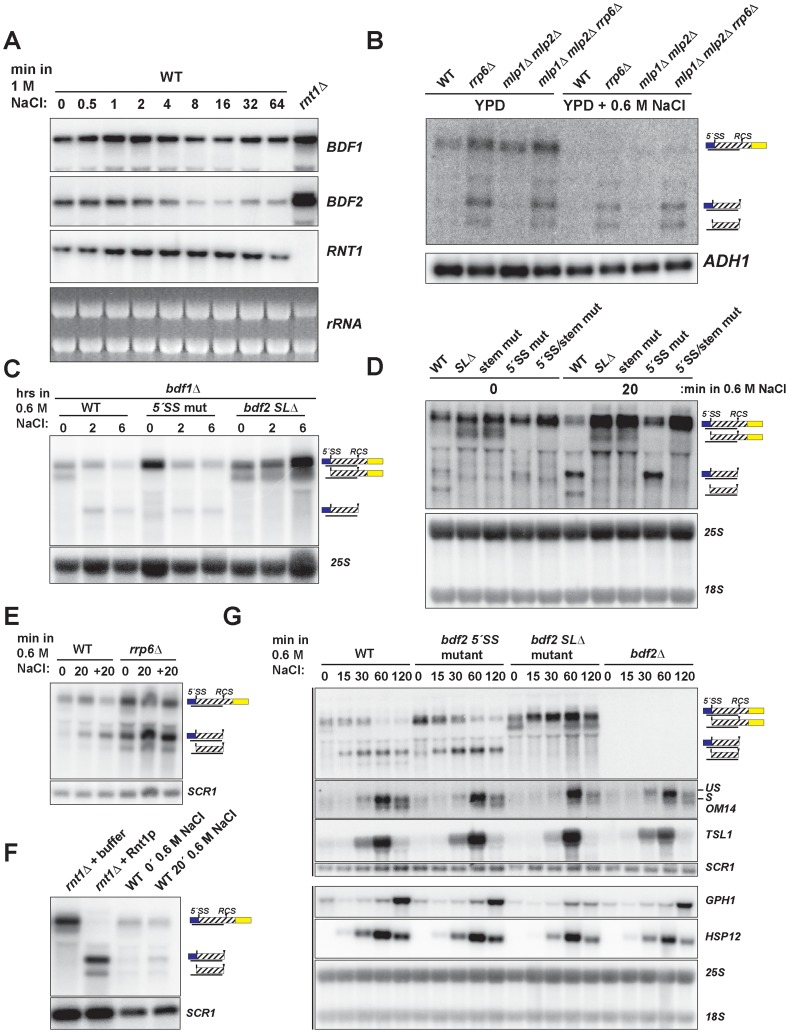
Hyper-activation of Rnt1p cleavage of *BDF2* mRNA in osmotic stress. **A**. The wild-type strain was exposed to high salt stress (1 M NaCl) and harvested at the indicated time points by dispensing cultures directly into −80°C pre-chilled ethanol. *BDF1* and *BDF2* were detected with riboprobes targeted to their respective 3′-UTRs. *RNT1* was detected with a riboprobe targeted to its open reading frame. A strain deleted for *RNT1* (*rnt1* ***Δ***) is included as a control (far right lane). The ethidium bromide-stained rRNAs are shown as loading controls. **B**. The indicated strains were grown in either normal conditions (YPD) or high salt conditions (YPD+0.6 M NaCl) under steady state growth. *BDF2* mRNA and its degradation intermediates were detected with a riboprobe targeting the exon1-intron region. *ADH1* is a loading control. **C**. *bdf1* ***Δ*** strains carrying either *WT BDF2*, the 5′-SS mutation (*5′-SS mut*), or the deletion of the Rnt1p target stem loop (*bdf2 SL* ***Δ***), were shifted from normal to high salt conditions for two and six hours. The 25S rRNA was detected with an oligonucleotide probe as a loading control. **D**. Wild-type *BDF1* strains with *WT BDF2, bdf2 SL* ***Δ***, the mutation of the stem-loop (*stem mut*), *5′-SS mut*, or both mutations (*5′-SS/stem mut*) were exposed to high salt for 20 minutes. The 25S and 18S rRNAs probed with radiolabeled oligonucleotides are shown as loading controls. **E**. WT and the nuclear exosome co-factor *RRP6* mutant (*rrp6* ***Δ***) were shifted for 20 minutes to 0.6 M NaCl (20), and then shifted back to normal medium for another 20 mintues (+20). *SCR1* is a loading control. **F**. RNA from the *rnt1* ***Δ*** strain was incubated with buffer or recombinant Rnt1p to demonstrate that the cleavage products observed in salt stress in the wild-type background co-migrate with the *in vitro* Rnt1p cleavage products. *SCR1* is a loading control. **G**. Wild-type and the specified *BDF2* mutants were shifted to 0.6 M NaCl for the time points indicated. An exon2 probe for the stress-induced, intron-containing *OM14* transcript detects both spliced and unspliced species. *TSL1*, *GPH1*, and *HSP12* were detected with riboprobes targeted to their respective open reading frames. Vertical black lines on the left side indicate panels that were part of the same blot. *SCR1* and the 25S and 18S rRNAs are loading controls.

The previous growth assays suggest that RMD plays an important role in regulating *BDF2* expression particularly in salt stress conditions. To understand how *BDF2* expression behaves in these conditions, we monitored *BDF2* mRNA levels in wild-type cells after a shift from normal to high salt medium. Within 8 minutes of a shift to high osmolarity, we observed a marked drop in the levels of the *BDF2* mRNA (Fig. 3A). By contrast, *BDF1* and *RNT1* mRNA levels increased in the first 8 minutes of osmotic shock. After 1 hour, *BDF2* levels were still repressed, while *BDF1* and *RNT1* levels had returned to pre-treatment levels. To assess *BDF2* expression and the levels of its RMD degradation intermediates after prolonged growth in high salt conditions, we monitored the levels of *BDF2* mRNA in wild-type and *rrp6Δ* strains during steady state growth in either normal medium or high salt (Fig. 3B). To test whether the Mlp1/2 factors might mediate increased nuclear retention and RMD of *BDF2* mRNA during osmotic stress, we also examined the *mlp1Δmlp2Δ* and *mlp1Δmlp2Δrrp6Δ* strains. Under normal growth conditions, we observed a stabilization of the 5′ product of RMD in the *rrp6Δ* strain, as expected (Fig. 3B). Remarkably, full-length *BDF2* was undetectable under steady-state growth in high-salt conditions, while the RMD cleavage products persisted. This suggested that transcription of the *BDF2* gene continues after prolonged exposure to osmotic stress, but that the transcripts are continuously degraded by SMD and RMD such that full-length *BDF2* mRNA is no longer detected. Inactivation of the Mlp1/2 factors had no effect on the levels of full-length *BDF2* mRNA or its degradation intermediates, suggesting that the increased targeting of *BDF2* mRNA by Rnt1p is independent of these nuclear retention factors.

To test whether the salt sensitivity of *bdf1Δ* is a consequence of increased RMD of *BDF2* mRNA, we examined *BDF2* mRNA levels in the *bdf1Δ* background after a prolonged shift to high salt conditions. This revealed a significant reduction of *BDF2* mRNA levels for the *WT BDF2* and the 5′-SS mutant after six hours (Fig. 3C). Notably, we detected the intron-exon2 product in *BDF2 WT* and the stem loop deletion strains in normal medium, as observed previously for mutations inactivating RMD in an otherwise wild-type *BDF1* background (Fig. 1F). This demonstrates that in normal growth conditions, spliceosome-mediated decay degrades a large fraction of *BDF2* mRNA in a manner that does not require Bdf1p, contrary to previous observations [13]. By two hours of shift to high salt, this SMD degradation intermediate disappeared and the RMD degradation intermediate was now detected in both the *WT* and the 5′-SS mutants. Strikingly, *BDF2* mRNA levels in the stem-loop deletion strain actually increased during this time course (Fig. 3C). This demonstrates that *BDF2* mRNA repression during osmotic stress is due to increased RMD rather than transcriptional repression. Furthermore, this confirms that *bdf1Δ*-salt sensitivity is a consequence of increased RMD and loss of *BDF2* full-length mRNA in osmotic stress, rather than a specific requirement for Bdf1p in activating osmotic stress response genes.

To examine how RMD affects the regulation of *BDF2* mRNA in the initial phase of the salt stress response, we monitored the levels of full-length *BDF2* mRNA and the SMD and RMD degradation products in the wild-type strain and strains carrying the 5′-SS and stem loop mutations after 20 minutes of osmotic shock (Fig. 3D). In normal medium, we detected the intron-exon2 product resulting from SMD in the stem-loop mutants. Significantly, we detected the accumulation of the RMD cleavage intermediate in WT and the 5′-SS mutant after 20 minutes in high salt (Fig. 3D). Consistent with the results from the *bdf1Δ* background, full-length *BDF2* mRNA increased in the stem-loop mutants, but not the 5′-SS mutant, suggesting that osmotic stress-induced transcripts are normally rapidly degraded by RMD. A previous study had found that the transcription rate of *BDF2* mRNA increases 10 minutes after osmotic shock in 0.4 M NaCl [42]. Our data show that this increase in transcription rate is counter-acted by an even greater increase in *BDF2* mRNA degradation through the RMD pathway.

To confirm that the increased abundance of the RMD cleavage intermediate is not due to a decrease in its degradation rate, we monitored levels of the cleavage product in the *rrp6Δ* strain, as the nuclear exosome is the primary factor involved in the decay of this cleavage intermediate. The RMD cleavage intermediate increased substantially after 20 minutes in osmotic shock in this context, showing that the increased abundance of this cleavage intermediate is due to hyper-activation of RMD (Fig. 3E). We confirmed that the *in vivo* cleavage bands induced in salt stress match the migration of the bands produced by *in vitro* Rnt1p cleavage (Fig. 3F). These results indicate that yeast RNase III plays a hitherto unappreciated role in the regulation of gene expression during osmotic stress.

We next tested the impact of an extended shift in 0.6 M NaCl on *BDF2* mRNA levels, and found a substantial reduction of full-length *BDF2* transcripts by one hour in both the WT and 5′-SS mutant (Fig. 3G). Strikingly, the *bdf2 SL* ***Δ*** mutant exhibited an increase in full-length *BDF2* mRNA levels throughout the first hour of high salt exposure, consistent with the increased transcription rate reported previously [42]. Interestingly, the SMD intron-exon2 product, present in *bdf2 SL* ***Δ*** in normal conditions, was absent after 15 minutes of salt stress (Fig. 3G). By one hour, this product re-appeared, suggesting that osmotic stress results in a transient deactivation of SMD (Fig. 3G). The dramatic decrease in *BDF2* mRNA levels detected in both the WT and the 5′-SS mutant, but not in the *bdf2 SL* ***Δ*** mutant, demonstrates that RMD is the primary mechanism controlling *BDF2* mRNA expression in salt shock conditions. The increase in RMD activity for the *BDF2* mRNA is not simply a consequence of decreased competition by SMD, resulting in increased flux of *BDF2* mRNA through RMD, because the 5′-SS mutation in normal salt conditions does not phenocopy the RMD hyper-activation that occurs during salt stress (Fig. 3G). Indeed, the *BDF2* mRNA harboring the 5′-SS mutation displays a similar profile in salt stress as WT *BDF2*, where by 60 minutes the RMD degradation product is present at higher levels than full-length *BDF2* mRNA (Fig. 3G). Overall, these data confirm that RMD of *BDF2* mRNA is hyper-activated in salt stress.

Next we investigated the impact of *BDF2* RMD on Bdf2p-regulated genes during osmotic stress. A previous study found that the loss of Bdf2p resulted in the upregulation of 20 transcripts at least 2-fold [10]. These transcripts were enriched in stress-responsive genes, including genes involved in carbohydrate metabolism and the heat shock response. We hypothesized that Bdf2p might normally repress these genes, and that an excess of Bdf2p, due to the inactivation of RMD, might inhibit the activation of these transcripts in response to osmotic stress. To test this hypothesis, we monitored the expression of various genes that were reported to be upregulated in *bdf2* ***Δ*** (Fig. 3G and S5). We found a significant decrease in the induction of the *GPH1* transcript in *bdf2 SLΔ* mutant after two hours, but not in the WT, *5′-SS* mutant, or *bdf2Δ* strains. *GPH1* encodes glycogen phosphorylase and is critical for preventing the over-accumulation of glycogen during various stress responses and in stationary phase [43]. Our data suggests that during osmotic stress, Rnt1p must repress *BDF2* mRNA levels in order for cells to fully induce *GPH1* expression.

### Inactivating the SMD of *BDF2* sensitizes the *bdf1Δ* mutant to DNA replication fork stress-inducing agents hydroxyurea and camptothecin

**Figure 4:**
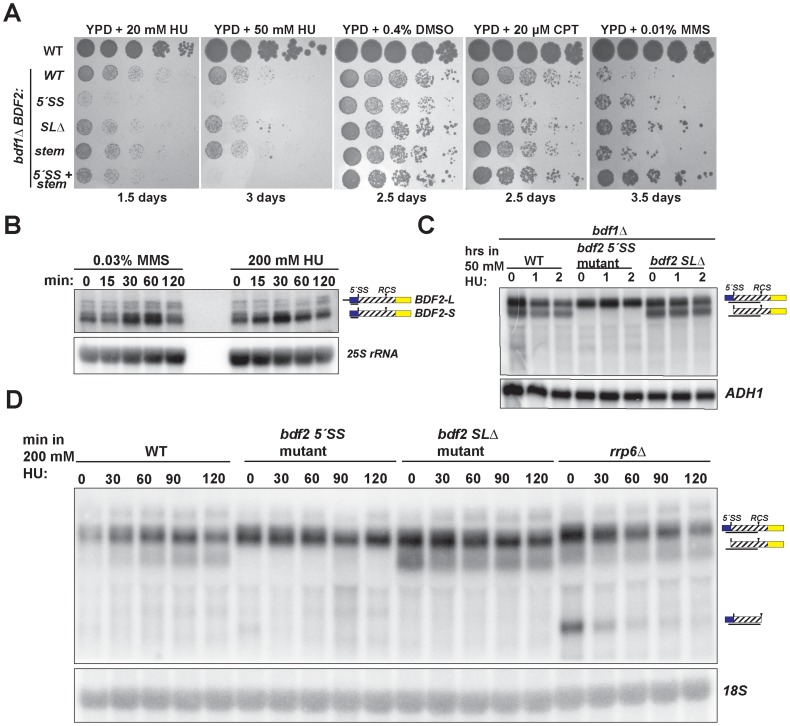
Spliceosome-mediated decay of *BDF2* mRNA predominates over Rnt1p cleavage during DNA replication stress. **A**. The set of strains from Figure 2 were grown on media containing the indicated concentrations of hydroxyurea (HU), dimethyl sulfoxide (DMSO), which was the vehicle control for camptothecin (CPT), and methyl methanesulfonate (MMS). **B**. The wild-type strain was incubated with the indicated concentrations of MMS or HU for the indicated time points. *BDF2* mRNA was detected with a riboprobe targeting exon1. The 25S rRNA is a loading control. **C**. *bdf1* ***Δ*** strains carrying either *WT BDF2*, the 5′-SS mutation (*5′-SS mut*), or the deletion of the Rnt1p target stem loop (*bdf2 SL* ***Δ***), were exposed to 50 mM HU for 1 and 2 hours. The *BDF2* full-length mRNA and SMD intron-exon2 degradation intermediates were detected with an exon1-intron riboprobe. *ADH1* is a loading control. **D**. The indicated strains were exposed to 200 mM HU for the specified time points. *BDF2* mRNA and the SMD and RMD degradation intermediates were detected with same probe as (C). The 18S rRNA was probed with a radiolabeled oligonucleotide as a loading control.

Cells lacking *BDF1* are hypersensitive to the DNA damage agents methyl methanesulfonate (MMS) and hydroxyurea (HU) [16]; [44]. The broad range of phenotypes rescued by inactivating RMD and SMD of *BDF2* mRNA led us to expect a rescue of *bdf1Δ* growth defects in DNA damaging conditions as well. Startlingly, inactivation of *BDF2* SMD resulted in enhanced toxicity of HU and camptothecin (CPT) for the *bdf1Δ* strain (Fig. 4A). Both HU and CPT result in the destabilization of replication forks and subsequent induction of double-strand breaks (DSBs) through different mechanisms [45], [46]. Interestingly, the stem loop mutation resulted in a slight rescue of the growth defect conferred by the 5′-SS mutation in 20 µM CPT, and to a lesser extent in 20 mM HU (Fig. 4A). It is possible that SMD and RMD predominate at different stages of the cell cycle, and in the presence of DNA replication stress, additional molecules of Bdf2p gained at one stage by inactivating RMD can compensate for the increased toxicity of excess Bdf2p due to SMD inactivation at another stage. Significantly, these results reveal that SMD plays an important role in the context of DNA replication stress. The lack of an effect from RMD inactivation in 50 mM HU led us to hypothesize that RMD activity on *BDF2* mRNA might be decreased in these conditions, and that the flux of *BDF2* mRNA degradation through SMD or RMD could be dependent upon specific environmental conditions.

### SMD predominates over RMD of *BDF2* mRNA in HU-induced DNA damage

The toxic effect of HU observed for the *bdf1Δ* strain harboring the 5′-SS mutation suggested that SMD protects against HU toxicity by preventing an excess accumulation of Bdf2p protein. Furthermore, the lack of toxicity of the stem-loop mutations indicated that SMD of the *BDF2* mRNA might predominate over RMD in DNA damage conditions. Bdf2p protein levels were reported to increase in response to the DNA damage agents MMS and HU [47]. To determine if the increase in protein is due to an increase at the mRNA level, we monitored *BDF2* mRNA expression after exposure to these DNA damage agents. Both treatments resulted in a transient increase in *BDF2* mRNA in the first hour, with slight differences in the kinetics, followed by a decrease to normal levels after two hours (Fig. 4B).

To further investigate how SMD and RMD affect the levels of *BDF2* mRNA in the *bdf1Δ* background, we exposed the 5′-SS and stem-loop mutants to 50 mM HU for two hours. There was no significant change in the levels of full-length *BDF2* mRNA or the intron-exon2 SMD degradation intermediate throughout this time course (Fig. 4C). Nonetheless, the time course demonstrated that SMD of *BDF2* mRNA remains active in the presence of HU. Because of the lack of an effect of the stem mutations on *bdf1Δ* growth in HU, we predicted that Rnt1p cleavage activity might decrease in HU. To test how RMD on *BDF2* mRNA behaves during HU exposure in the wild-type *BDF1* background, we treated wild-type, the 5′-SS mutant, the stem-loop deletion mutant, and *rrp6Δ* strains with 200 mM HU and monitored the expression of *BDF2* mRNA and its degradation products. Consistent with reduced RMD, there was a substantial drop in the levels of the RMD intermediate within 30 minutes of HU treatment in the *rrp6Δ* background (Fig. 4D). Furthermore, the intron-exon2 SMD product, which is normally degraded by Rnt1p due to the presence of the Rnt1p target stem loop in the intron, actually increased in the WT strain throughout the HU treatment. Together these results indicate that RMD of *BDF2* mRNA is inhibited during DNA replication stress, and that the increased flux of *BDF2* mRNA through SMD is important to protect *bdf1Δ* cells from the toxic effects of excess Bdf2p accumulation.

## Discussion

### *BDF2* expression is predominantly regulated at the level of RNA decay

In this study we demonstrate that two major nuclear RNA degradation pathways, Rnt1p- and spliceosome-mediated decay, limit the expression of *BDF2* mRNA, and that *BDF2* expression is tightly regulated at the post-transcriptional level in specific environmental conditions. Strikingly, in normal growth conditions, the *BDF2* gene exhibits a high transcription rate (0.51 RNA molecules per minute per cell) in the top 4.6% of all RNA polymerase II (Pol II)-transcribed genes [29], with an mRNA stability that is among the lowest of all *S.cerevisiae* Pol II transcripts (∼5 minute half life, lowest 1.1% [48]). During osmotic stress, a condition where *BDF2* mRNA levels rapidly drop, the Pol II transcription rate across the *BDF2* gene paradoxically increases. However, RNase III-mediated degradation (RMD) over-compensates for the increased transcription, and *BDF2* mRNA levels drop rapidly. Simultaneously, there is a transient decrease in spliceosome-mediated decay (SMD) of *BDF2* mRNA during salt stress. A decrease in spliceosome activity during osmotic stress is consistent with a recent report that intron-containing ribosomal protein pre-mRNAs, the predominant substrates for the spliceosome, accumulate as unspliced transcripts in osmotic stress conditions when their degradation by nonsense-mediated decay is inactivated [49]. Therefore, both nuclear and cytoplasmic RNA degradation systems have evolved to degrade intron-containing transcripts that accumulate during osmotic stress. By contrast, SMD of *BDF2* mRNA plays a critical role during DNA replication stress, as evidenced by the toxicity of the 5′-SS mutation to cells lacking *BDF1* when grown in the presence of DNA double-strand break agents (Fig. 4A). Conversely, we found that RMD of *BDF2* mRNA is diminished during hydroxyurea exposure and its inactivation confers no phenotype towards *bdf1Δ* cells in HU or CPT (Fig. 4A,D). Interestingly, there was not a significant effect of the 5′-SS mutation on *BDF2* mRNA levels in HU during the time window we examined (Fig. 4C,D). It is possible that an effect of HU on *BDF2* mRNA levels in the 5′SS mutant requires longer exposure times to HU. It is also possible that the 5′SS mutation results in enhanced leakage of the “unspliced-like” *BDF2* mRNA to the cytoplasm and thus increased levels of Bdf2p translation, as the nuclear retention of intron-containing transcripts by Mlp1p is known to require an intact 5′-splice site [38]. This would be consistent with our finding that Mlp1/2p proteins regulate *BDF2* mRNA levels (Fig. S4B).

**Figure 5:**
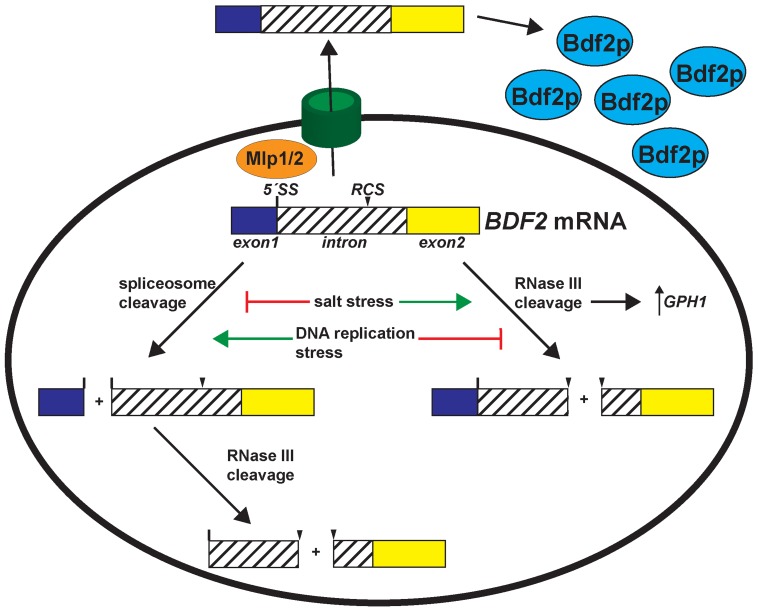
Environmental stress conditions control the expression of Bromodomain Factor 2 mRNA through modulating RNase III and spliceosome-mediated decay. Salt stress transiently deactivates spliceosome-mediated decay and hyper-activates RNase III cleavage of *BDF2*, which is required for the full induction of the stress-induced *GPH1* transcript. DNA replication stress de-activates RNase III cleavage of *BDF2* mRNA, while spliceosome-mediated decay persists in these conditions to repress *BDF2* expression. In addition, the Mlp1/2 nuclear retention factors repress *BDF2* expression. *BDF2* mRNAs that escape nuclear surveillance are exported and translated.

Our results reveal that the relative flux of *BDF2* mRNA degradation through RMD or SMD is dependent on the particular environmental condition, such that when one degradation system is inactivated, the other system predominates (Fig. 5). In particular, we found that levels of the SMD degradation intermediate decrease in salt stress, whereas the RMD intermediate increases in abundance. During DNA replication stress, the RMD intermediate decreases while the SMD degradation intermediate increases. These molecular phenotypes are consistent with the growth phenotypes of the *BDF2* 5′-SS and *BDF2* stem loop mutations in the *bdf1Δ* background in these stress conditions. It appears that the *BDF2* gene has evolved to retain the degradation signals for both systems, such that its tight post-transcriptional control is maintained in conditions that block either RMD or SMD. Therefore, the presence of two independent nuclear degradation pathways acting on the *BDF2* mRNA guards against excess accumulation of *BDF2* mRNA across a range of stress conditions. The observation that *BDF2* mRNA is detectable in WT cells in normal growth conditions suggests that a substantial fraction of *BDF2* mRNA normally escapes nuclear degradation by SMD and RMD. In salt stress, an increase in the absolute activity of RMD leads to a substantial increase in the overall fraction of full-length *BDF2* mRNA subject to nuclear degradation. Furthermore, inhibiting both RMD and SMD has an additive effect compared to inhibiting either pathway alone in specific conditions (Fig. 1D and Fig. 2B), demonstrating that the pathways are non-redundant and independently regulate *BDF2* mRNA in a condition-specific manner (Fig. 5).

### RMD and SMD modulate Bromodomain factor 2 expression to maintain cellular fitness in response to adverse environmental conditions

Despite detailed biochemical work on the binding affinities of Bdf1p and Bdf2p for different acetylated histone tail peptides [50], and extensive chromatin immunoprecipitation studies mapping genomic binding sites [8], it is largely unknown how Bdf1p and Bdf2p achieve their specificity *in vivo* and how these factors are regulated in different conditions. We found that during osmotic stress, degradation of *BDF2* mRNA is required for the full induction of glycogen phosphorylase (*GPH1*, Fig. 3F), suggesting that Bdf2p may normally repress a subset of salt-stress induced genes. We also found that inactivating SMD or RMD in a wild-type background resulted in sensitivity to lithium chloride (Fig. S6), suggesting that decreasing *BDF2* mRNA levels via nuclear RNA decay constitutes an important mechanism for adapting gene expression to lithium ion stress.

The regulation of *BDF2* expression by these two RNA degradation mechanisms controls highly specific phenotypes. Strikingly, the inactivation of SMD, but not RMD, sensitized *bdf1Δ* cells to hydroxyurea (HU) and camptothecin (CPT), but not methyl methanesulfonate (MMS) (Fig. 4A). While all three agents stall replication fork progression, only HU and CPT induce *in vivo* double-strand DNA breaks (DSBs) [51]. This suggests that an excess of Bdf2p in the absence of Bdf1p is toxic specifically in the presence of DSBs. This finding is consistent with a report that the fission yeast homolog of *BDF2* is toxic to cells with unstable replication forks, as the deletion of *BDF2* suppressed the HU sensitivity of numerous S-phase checkpoint mutants [17]. It remains to be determined whether the toxicity of Bdf2p is due to a direct effect on chromatin structure, or rather due to its effects on gene expression in DNA damage conditions. Notably, the human homolog of Bdf2p, *BRD4*, has been shown to activate cell death in response to DNA damage by promoting chromatin condensation [52]. It is therefore possible that at least some of the functions of Bdf2p in the DNA damage response have been conserved from yeast to humans. Significantly, we found that *BDF2* is required for resistance of wild-type cells to DMSO (Fig. S6), which has been implicated in causing DNA damage by an unknown mechanism [53]. Therefore, Bdf2p is beneficial in specific conditions, and can play antagonizing roles in the DNA damage response depending on the specific type of DNA damage.

Overall, our study highlights how environmental stress conditions can differentially control RNA degradation systems to regulate gene expression (Fig. 5), and emphasizes the importance of modulating bromodomain factors expression in the cellular response to environmental changes. The ability of the cell to degrade or stabilize specific transcripts by environmentally-controlled RNA degradation pathways mirrors the control that transcriptional activators and repressors can provide in response to stress conditions. We speculate that the presence of SMD and/or RMD degradation signals in other mRNAs may allow for broad post-transcriptional fine-tuning of gene expression in conditions where the activity of these degradation systems is modulated. Future work will unravel precisely what mechanisms regulate RNase III and spliceosome-mediated decay in different environmental conditions, as well as uncover the repertoire of transcripts targeted by these pathways.

## Materials and Methods

### RNA secondary structure screen

Transcripts targeted by spliceosome-mediated decay were obtained from Supplementary Table 2 from [13]. The 5′ and 3′ UTRs for each transcript were obtained from [31] from *Saccharomyces Genome Database*. If the transcript did not have an annotated UTR, the open reading frame start or stop coordinates were used. Each sequence was first split into 200 base fragments in 100 base overlapping steps. mFold [24] was used to predict the fold of each 200 base sequence, and sequences capable of adopting stem-loop structures with an NGNN or AAGU tetraloop were identified for further study.

### Strains

The BMA64 (mating type a) background was used to generate the mutant strains used in this study (Table S1). The indicated deletion mutants were constructed by the lithium acetate/PEG method [54] by replacing the open reading frames with PCR products encoding either *S. cerevisiae TRP1, S. kluyveri HIS5*, or the *KANMX6* gene [55], [56]. Strains containing the *rat1-1* mutation were previously described [28]. Mutations were introduced into the endogenous *BDF2* gene by the *delitto perfetto* method [57]. First, the CORE cassette encoding the *URA3* and *KANMX6* genes was inserted at Rnt1p-target stem loop (chrIV:332367-80). G418 resistant colonies were screened for successful integration of the CORE cassette by PCR. For the second step, we used the pUG23-*BDF2* plasmid (described below), containing either the WT or various *BDF2* mutations, as a template to generate high-fidelity PCR products (NEB Phusion). Primers were designed so that the resulting PCR products contained >100 bases of homology flanking the mutation sites (Table S1). 200 µl of each PCR product were precipitated in 1 ml ethanol and 40 µl 3 M sodium acetate pH 5.2. Pellets were washed in 70% ethanol and resuspended in 34 µl water and used for excision of the CORE cassette by standard yeast transformation techniques [54]. Transformations were plated overnight on YPD, and then replica-plated to 5-FOA to select for loss of *URA3*. Colonies were screened for sensitivity to G418 to confirm loss of the CORE cassette, and mutations were confirmed by PCR amplification of the *BDF2* locus followed by sequencing (Laragen, Inc).

### Plasmids

The *BDF2* open reading frame without the stop codon was amplified by high-fidelity PCR (NEB Phusion) from WT genomic DNA with the forward primer containing the SpeI restriction site and the reverse primer containing the ClaI restriction site (Table S1). The insert was cloned into the pUG23 vector and 5′-splice site and stem-loop mutations were constructed with the Quick-Change site-directed mutagenesis kit (Agilent Technologies, Inc). All plasmids and mutations were confirmed by sequencing (Laragen, Inc.).

### Media and culturing

Unless otherwise indicated, cultures were grown in YPD (1% yeast extract, 2% bacto-peptone, 2% dextrose) at 30°C at 200 rpm. SDC (.67% yeast nitrogen base with ammonium sulfate, 2% dextrose, and 0.2% complete amino acid mixture) was used to culture strains containing the *met22Δ* deletion, and where indicated these cultures were pelleted, washed once with SD medium lacking methionine (SD-MET), and resuspended in SD-MET for 12 hours. Strains containing the pUG23 vector were grown in SD-URA-MET. Strains containing the *rat1-1* mutation were grown at 25°C and shifted to pre-warmed flasks containing the same medium at 37°C. All cultures were harvested by centrifugation at 4000 rpm for 2 minutes, washed in deionized water, and spun down in microcentrifuge tubes. The supernatant was removed and pellets were flash-frozen in liquid nitrogen and stored at −80°C.

### RNA extraction and northern blot analysis

RNA extractions were performed by the addition of 400 µl acid-washed glass beads, 500 µl phenol∶chloroform∶isoamyl alcohol (25∶24∶1, pH 6.7, Fisher Scientific), and 500 µl of RNA-SDS buffer (50 mM Tris-HCl pH 7.5, 100 mM NaCl, 10 mM EDTA, 2% SDS) to the frozen pellets. Samples were vortexed for 1 minute, heated in a 65°C water bath for 5 minutes, and vortexed for an additional minute. Samples were spun at 13,200 rpm for 5 minutes, and 450 µl of the supernatant was transferred to a new tube containing 450 µl of phenol∶chloroform∶isoamyl alcohol. Samples were vortexed for 1 minute, and spun at 15,000 rpm for 5 minutes. 400 µl of supernatant were precipitated in 1 ml EtOH and 40 µl of 3M NaOAc pH 5.2, and incubated at −80°C for 30 minutes. Samples were spun at 15,000 rpm for 5 minutes, and pellets were washed with 70% EtOH and resuspended in nuclease-free water (Ambion). *In vitro* cleavage of 50 µg total RNA with 4 pmol recombinant Rnt1p was performed at 30°C for 20 minutes as described [58], [59]. 1 volume of RNA was denatured in 5 volumes of glyoxal buffer [60% DMSO (Sigma-Aldrich), 8% glyoxal w/v (Sigma-Aldrich), 5% glycerol, 40 µg/ml ethidium bromide, 1× BPTE pH 6.5 (10 mM PIPES (Sigma-Aldrich), 30 mM Bis-Tris (Sigma-Aldrich), 10 mM EDTA pH 8.0)]. Samples were vortexed and denatured at 55°C for one hour. Samples were chilled on ice for 10 minutes, and 10× BPTE-RNA loading dye (0.25% xylene cyanol, 0.25% bromophenol blue, 5% glycerol, 1× BPTE) was added to each sample before loading on 2% agarose gels with 1× BPTE buffer. Electrophoresis was performed at 4 V/cm while mixing the buffer with magnetic stir bars. Gels were visualized and washed for 10 minutes in deionized water, 20 minutes in 75 mM NaOH, an additional 10 minutes in deionized water, and 10 minutes in 10× SSPE (1.5 M NaCl, 100 mM sodium phosphate, 100 mM EDTA, pH 7.4). Gels were passively transferred in 10× SSPE to positively charged nylon membranes (Hybond-N^+^, GE Healthcare Life Sciences). Blots were cross-linked with the auto cross-link function (120,000 joules/cm^2^) on the Stratalinker UV Crosslinker 2400 (Stratagene), and stored in 2× SSPE at 4°C. RNA probes were constructed by *in vitro* transcription with the T3 RNA polymerase (Promega) and α-^32^P- UTP (Perkin-Elmer) following the manufacturer's protocol, with the exception that radioactive UTP was used instead of radioactive CTP. Templates were constructed by PCR using genomic DNA and primers hybridizing to the target gene (Table S1). The reverse primer contained at its 5′-end the T3 promoter sequence (5′-AATTAACCCTCACTAAAGGGA-3). Blots were pre-hybridized in Church's buffer (1% BSA, 1 mM EDTA, 0.5 M NaPO_4_ pH 7.2, 7% SDS) at 65°C for 1 hour, and transcription reactions were diluted in 100 µl of deionized water and added directly to the hybridization bottles. Hybridizations were conducted overnight at 65°C, and blots were washed for 20 minutes with 2× SSPE+0.1% SDS at 65°C, followed by two washes for 20 minutes with 0.1× SSPE+0.1% SDS at 65°C. Blots were exposed to K-screens (Kodak) and scanned with the BioRad FX Phosphorimager.

### Primer extension mapping of the Rnt1p cleavage site

20 pmol of *BDF2* downstream RCS reverse primer (5′-CAATTGTTGCAATTCAACCTCC-3′) was labeled with γ-^32^P-ATP with T4 polynucleotide kinase (NEB) according to the manufacturer's protocol. Primer extension was performed using the SuperScript III reverse transcriptase (Invitrogen/Life Technologies) according to manufacturer's protocol, except that denaturation was performed at 75°C for 4 minutes in a thermocycler, followed by primer annealing at 45°C for 5 minutes. Primer extension was performed at 55°C for 60 minutes, followed by 70°C for 15 minutes to inactivate the enzyme. An equal volume of 2× formamide loading dye (Ambion) was added to the primer extension reactions, heated at 75°C for 3 minutes, and 10 µl was loaded onto 8% poly-acrylamide sequencing gels. To obtain the sequencing ladder, a PCR product was first generated using the reverse primer and the *BDF2* exon1 forward primer (5′-GCACATTCTGCTTTACTGGCAGC-3′) and pUG23-*BDF2* as template. The PCR product was used as a template with the Thermo Sequenase Cycle Sequencing Kit using 0.5 picomoles of labeled primer for each of the 4 dideoxy-nucleotide sequencing reactions (according to manufacturer's protocol). Gels were dried at 80°C for one hour on a vacuum gel drier (Amersham Pharmacia), and exposed to K-screens (Kodak) and scanned with the BioRad FX Phosphorimager.

### RT-PCR analysis

40 µg total RNA of total RNA was treated with DNase I (Ambion) according to manufacturer's protocol. 5 µg of DNase I-treated RNA was reverse-transcribed with random hexamers and M-MLV reverse transcriptase (Invitrogen) according to manufacturer's protocol. 2 µl of cDNA was used as a template in standard 50 µl PCR with the forward primer 5′-AGCAGCGAGAGTAGTAGTAACAAAAAC-3′ and either reverse primer R1 5′-ATCTCTCATAAATTCTTGCAATAGTCG-3′ or R2 5′-TGCTTCATTGCCTTCCTACG-3′. The following thermo-cycler parameters were used: 94°C 2 min, 35 cycles of 94°C 30 s, 60°C 30 s, 72°C 60 s. RT-PCR products were analyzed on 1× TAE 2% agarose gels and stained with ethidium bromide. The identity of each band was verified by gel extraction of the excised band followed by Sanger sequencing (Laragen, Inc.).

### Plate growth assays

Cultures were grown to mid-log phase (OD_600 nm_ 0.4–0.6) and 1 ml of each culture was spun at 13,200 rpm for 30 seconds. Pellets were resuspended in 1 ml of sterile water, and diluted to OD_600 nm_ 0.1 followed by four 5-fold serial dilutions in sterile water. 4 µl of each dilution was spotted onto the indicated media. Hydroxyurea was purchased from Alfa-Aesar, and camptothecin and methyl methanesulfonate were purchased from Sigma-Aldrich.

## Supporting Information

Figure S1 Northern blot analysis of genes identified as targets of spliceosome-mediated decay (Supp. Table 2 from ([13]) containing RNA sequences capable of folding into canonical Rnt1p target stem-loops. Riboprobes for the indicated genes were designed to target the open reading frames. *SCR1* is shown as a loading control. The *NPL3* readthrough transcript is a positive control for a known Rnt1p mRNA target [60], [61].
(TIF)

Figure S2 RT-PCR analysis of full-length *BDF2* mRNA and spliced products generated by spliceosome-mediated decay in wild-type, the strain carrying the disruption of the Rnt1p stem loop (*bdf2 stem mutant*), and the deletion of nuclear exosome co-factor *RRP6* (*rrp6* ***Δ***). (A) The locations of the forward (F) and reverse primers (R1 and R2) are shown relative to the Rnt1p cleavage site (*RCS*), the previously annotated AAG 3′-splice site at +1672 into the ORF [13], and an alternative AAG 3′-splice site at +1595 (identified here by sequencing). The stem mutant introduces two 3′-splice site CAG motifs into the Rnt1p target stem-loop downstream of the (UUC)_3_ polypyrimidine tract. (B) RT-PCR was performed with the same forward primer and either R1 (top panel) or R2 (bottom panel). The spliced species corresponding to each band is indicated to the right of each band. The band denoted with an asterisk in both panels arises from mis-priming of the F primer 3′end at +1426 in the *BDF2* ORF.
(TIF)

Figure S3 Probe walking to verify the identity of the *BDF2* mRNA degradation intermediates generated by spliceosome-mediated decay and Rnt1p. RNA from the indicated strains was analyzed on the same gel in a triplicate series and transferred to three strips of membranes. Riboprobes were designed to hybridize to exon1 (probe I), the intron (probe II), or exon2 (probe III) of *BDF2* mRNA and blots were aligned during the exposure to compare the migration of each *BDF2* species. The band labeled with the asterisk on the exon1 blot is due to cross-hybridization with the 18S rRNA.
(TIF)

Figure S4 The nuclear exosome co-factor *RRP6*, the nonsense mediated decay (NMD) factor *UPF1*, and the pre-mRNA nuclear retention factors *MLP1/2* participate in the surveillance of *BDF2* transcripts. (**A**) A riboprobe for the *BDF2* exon1 detects the 5′ products of Rnt1p and spliceosome endonucleolytic cleavage (left panel). A riboprobe was designed to hybridize to the 5′ UTR of *BDF2-L*, upstream of the annotated transcription start site for *BDF2-S* (chrIV: 331002), in order to exclusively detect *BDF2-L* and its RMD degradation intermediate (right panel). (**B**) A riboprobe spanning exon 1 and the intronic region up to the Rnt1p cleavage site (*RCS*) (+23 to +1303 into the ORF) detects full-length *BDF2* as well as Rnt1p cleavage products (top half). The *BDF2-L* full-length transcript and its RMD product were detected with the 5′-UTR probe that does not bind *BDF2-S* (bottom half).
(TIF)

Figure S5 Wild-type and the specified *BDF2* mutants were shifted to 0.6 M NaCl for the time points indicated. *GLK1, HAL5, GRE3*, and *LEU1* were detected with riboprobes targeting the open reading frames of their respective transcripts. SCR1 is a loading control.
(TIF)

Figure S6 The wild-type strain and the specified *BDF2* mutants were grown on plates with the indicated media at 30°C for the indicated number of days. (**A**) Inactivation of RMD or SMD of *BDF2* mRNA has no effect on the growth of the wild-type background in high salt or hyperosmotic stress conditions. (**B**) Inactivation of RMD or SMD of *BDF2* mRNA confers sensitivity to 0.6 M lithium chloride, but not methyl methanesulfonate (MMS) or hydroxyurea (HU). (**C**) Cells lacking *BDF2* are hyper-sensitive to 2% dimethyl sulfoxide (DMSO), and do not grow on the combination of 2% DMSO and 100 µM camptothecin (CPT).
(TIF)

Table S1 *Saccharomyces cerevisiae* strains, plasmids, and oligonucleotides used in this study. The strain ID, genotype and reference is provided for each strain studied. The plasmids are generated from the pUG23 vector, and contain the described mutations in the *BDF2* ORF. The oligonucleotide sequences used to generate T3 riboprobe templates or radiolabeled oligonucleotide probes are indicated, with a reference to the relevant figures. The oligonucleotides used to generate the plasmids and yeast strains are indicated along with the purpose of each primer pair.
(XLSX)
