# Supplementary figures and images for: Stress-Induced Nuclear RNA Degradation Pathways Regulate Yeast Bromodomain Factor 2 to Promote Cell Survival
Source: PLoS Genet. 2014 Sep 18;10(9):e1004661. doi: 10.1371/journal.pgen.1004661 (PMC4169253; doi:10.1371/journal.pgen.1004661)

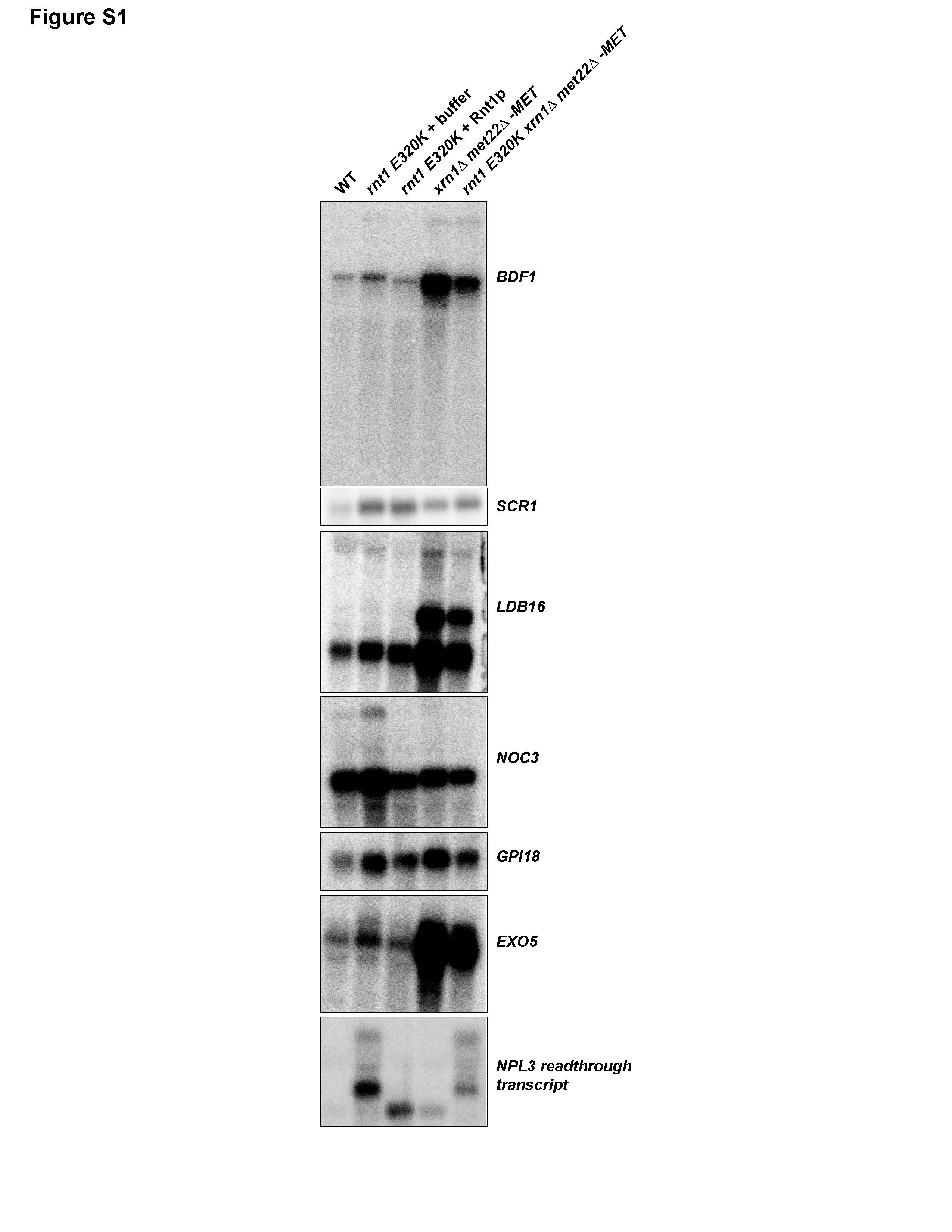

Supplement: Figure S1 — Northern blot analysis of genes identified as targets of spliceosome-mediated decay (Supp. Table 2 from ([13]) containing RNA sequences capable of folding into canonical Rnt1p target stem-loops. Riboprobes for the indicated genes were designed to target the open reading frames. SCR1 is shown as a loading control. The NPL3 readthrough transcript is a positive control for a known Rnt1p mRNA target [60], [61]. (TIF) [file pgen.1004661.s001.tif]

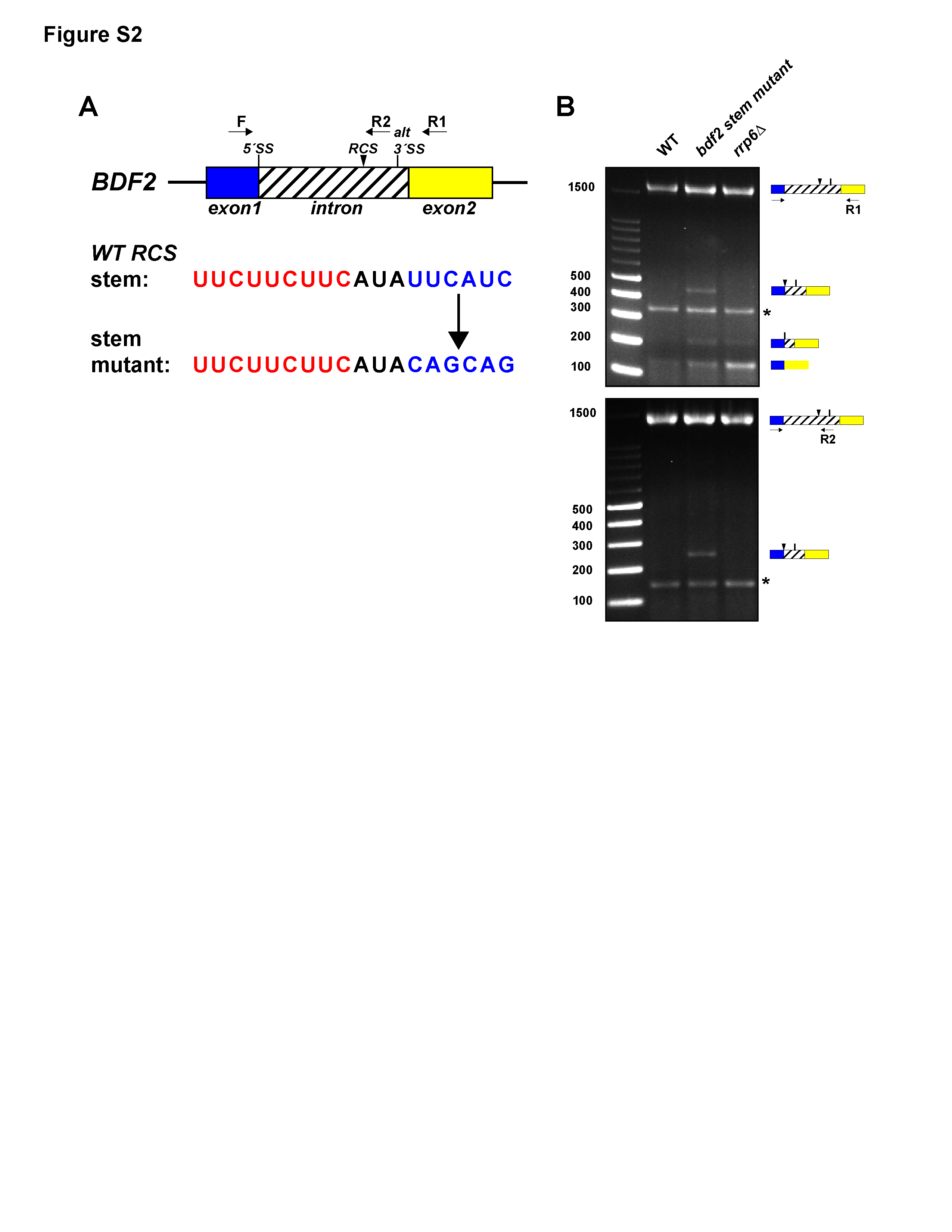

Supplement: Figure S2 — RT-PCR analysis of full-length BDF2 mRNA and spliced products generated by spliceosome-mediated decay in wild-type, the strain carrying the disruption of the Rnt1p stem loop (bdf2 stem mutant), and the deletion of nuclear exosome co-factor RRP6 (rrp6 Δ). (A) The locations of the forward (F) and reverse primers (R1 and R2) are shown relative to the Rnt1p cleavage site (RCS), the previously annotated AAG 3′-splice site at +1672 into the ORF [13], and an alternative AAG 3′-splice site at +1595 (identified here by sequencing). The stem mutant introduces two 3′-splice site CAG motifs into the Rnt1p target stem-loop downstream of the (UUC)3 polypyrimidine tract. (B) RT-PCR was performed with the same forward primer and either R1 (top panel) or R2 (bottom panel). The spliced species corresponding to each band is indicated to the right of each band. The band denoted with an asterisk in both panels arises from mis-priming of the F primer 3′end at +1426 in the BDF2 ORF. (TIF) [file pgen.1004661.s002.tif]

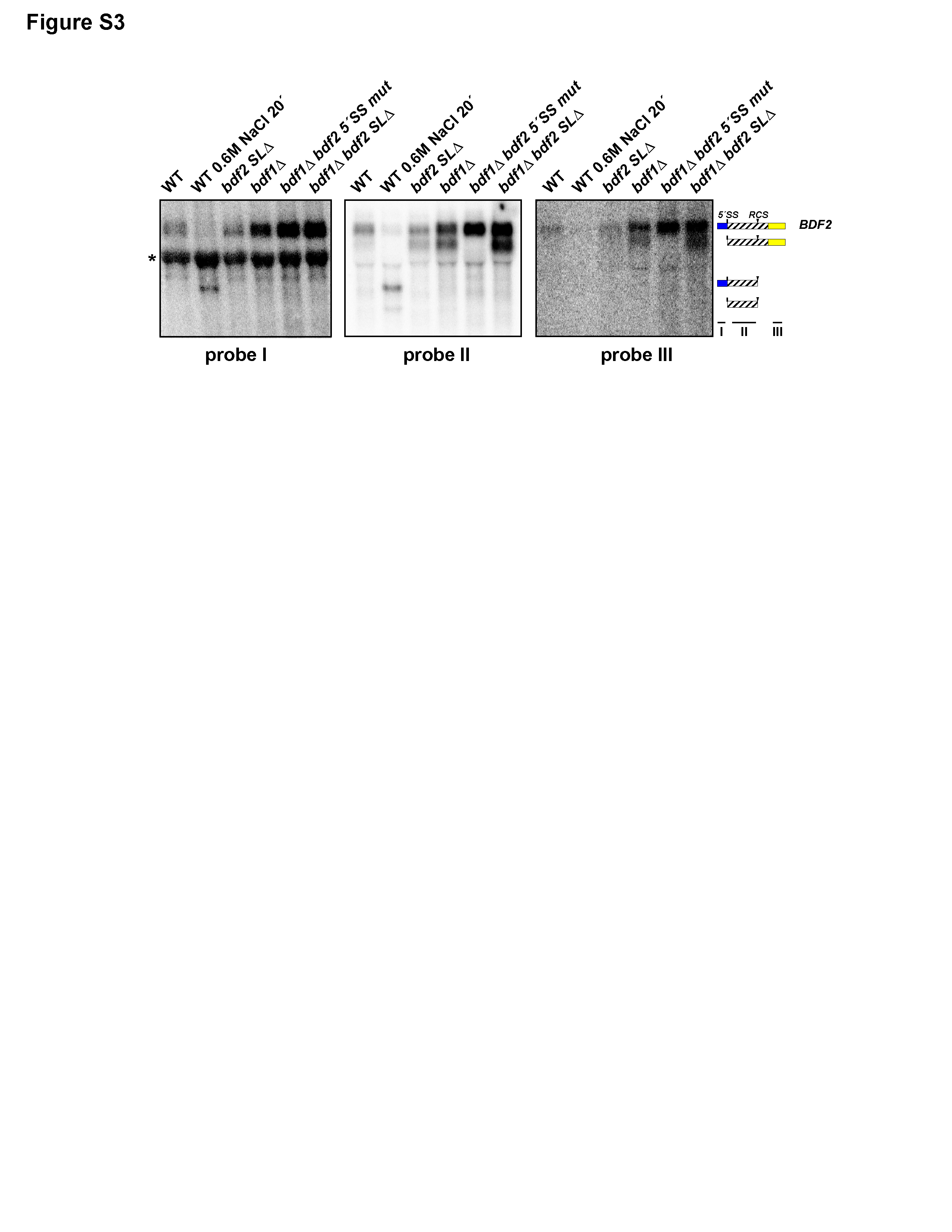

Supplement: Figure S3 — Probe walking to verify the identity of the BDF2 mRNA degradation intermediates generated by spliceosome-mediated decay and Rnt1p. RNA from the indicated strains was analyzed on the same gel in a triplicate series and transferred to three strips of membranes. Riboprobes were designed to hybridize to exon1 (probe I), the intron (probe II), or exon2 (probe III) of BDF2 mRNA and blots were aligned during the exposure to compare the migration of each BDF2 species. The band labeled with the asterisk on the exon1 blot is due to cross-hybridization with the 18S rRNA. (TIF) [file pgen.1004661.s003.tif]

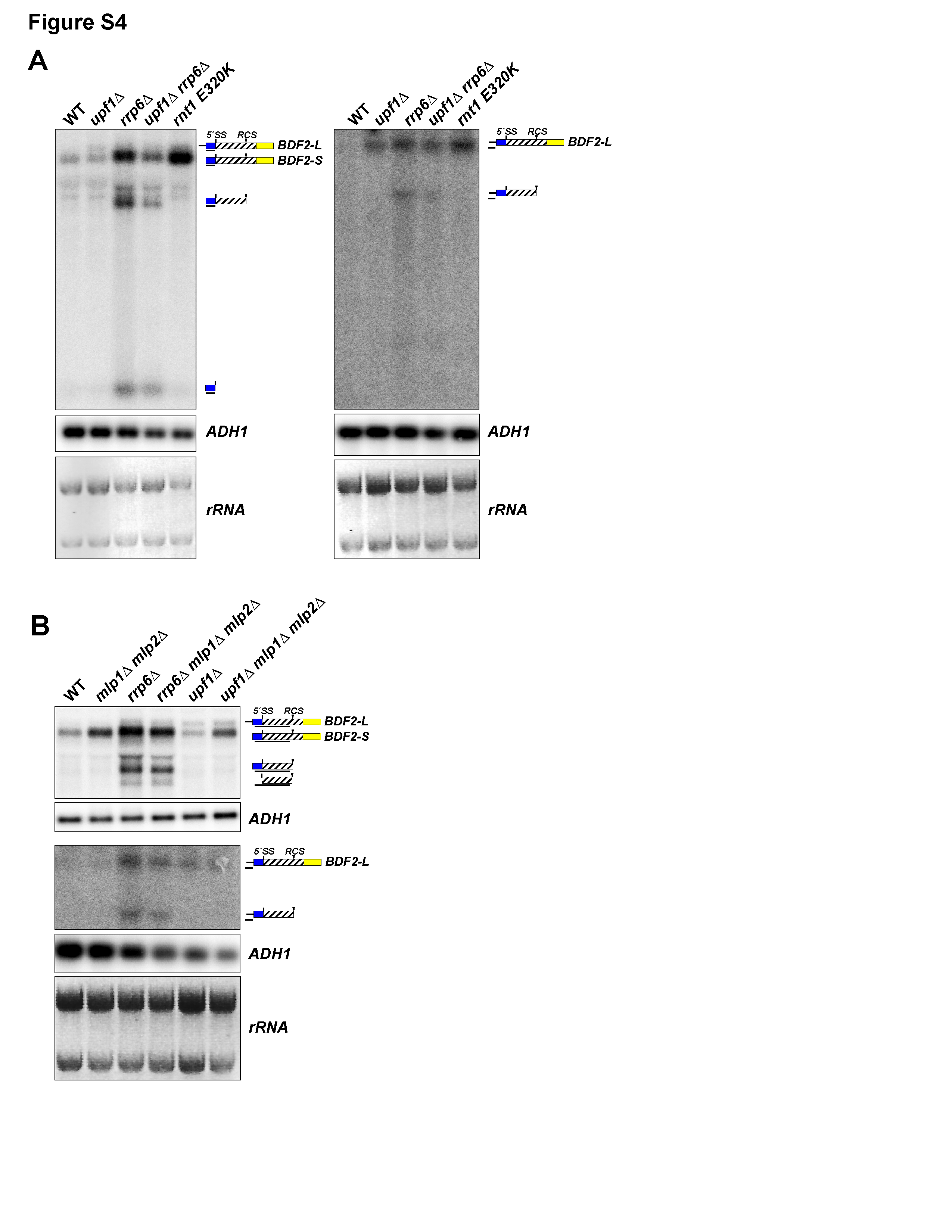

Supplement: Figure S4 — The nuclear exosome co-factor RRP6, the nonsense mediated decay (NMD) factor UPF1, and the pre-mRNA nuclear retention factors MLP1/2 participate in the surveillance of BDF2 transcripts. (A) A riboprobe for the BDF2 exon1 detects the 5′ products of Rnt1p and spliceosome endonucleolytic cleavage (left panel). A riboprobe was designed to hybridize to the 5′ UTR of BDF2-L, upstream of the annotated transcription start site for BDF2-S (chrIV: 331002), in order to exclusively detect BDF2-L and its RMD degradation intermediate (right panel). (B) A riboprobe spanning exon 1 and the intronic region up to the Rnt1p cleavage site (RCS) (+23 to +1303 into the ORF) detects full-length BDF2 as well as Rnt1p cleavage products (top half). The BDF2-L full-length transcript and its RMD product were detected with the 5′-UTR probe that does not bind BDF2-S (bottom half). (TIF) [file pgen.1004661.s004.tif]

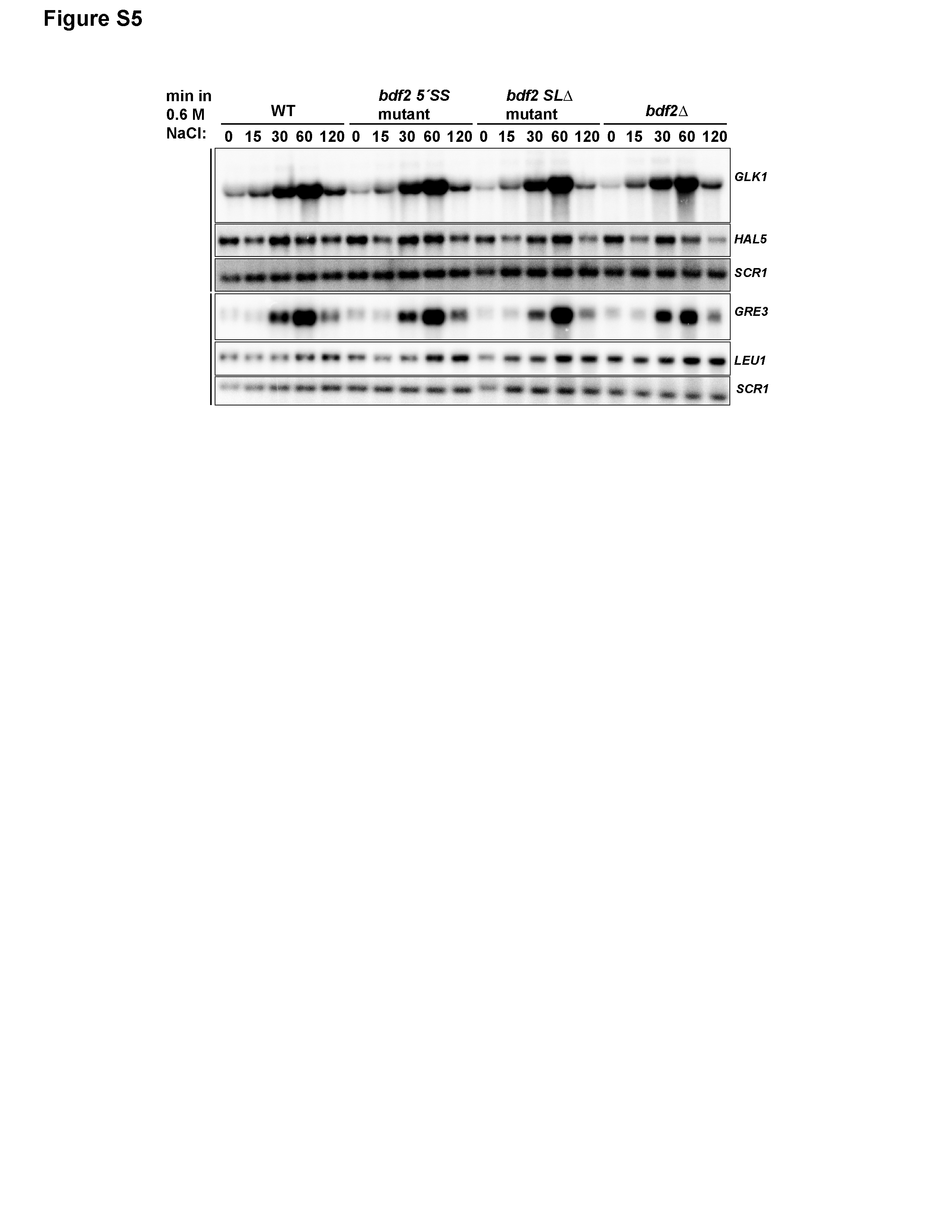

Supplement: Figure S5 — Wild-type and the specified BDF2 mutants were shifted to 0.6 M NaCl for the time points indicated. GLK1, HAL5, GRE3, and LEU1 were detected with riboprobes targeting the open reading frames of their respective transcripts. SCR1 is a loading control. (TIF) [file pgen.1004661.s005.tif]

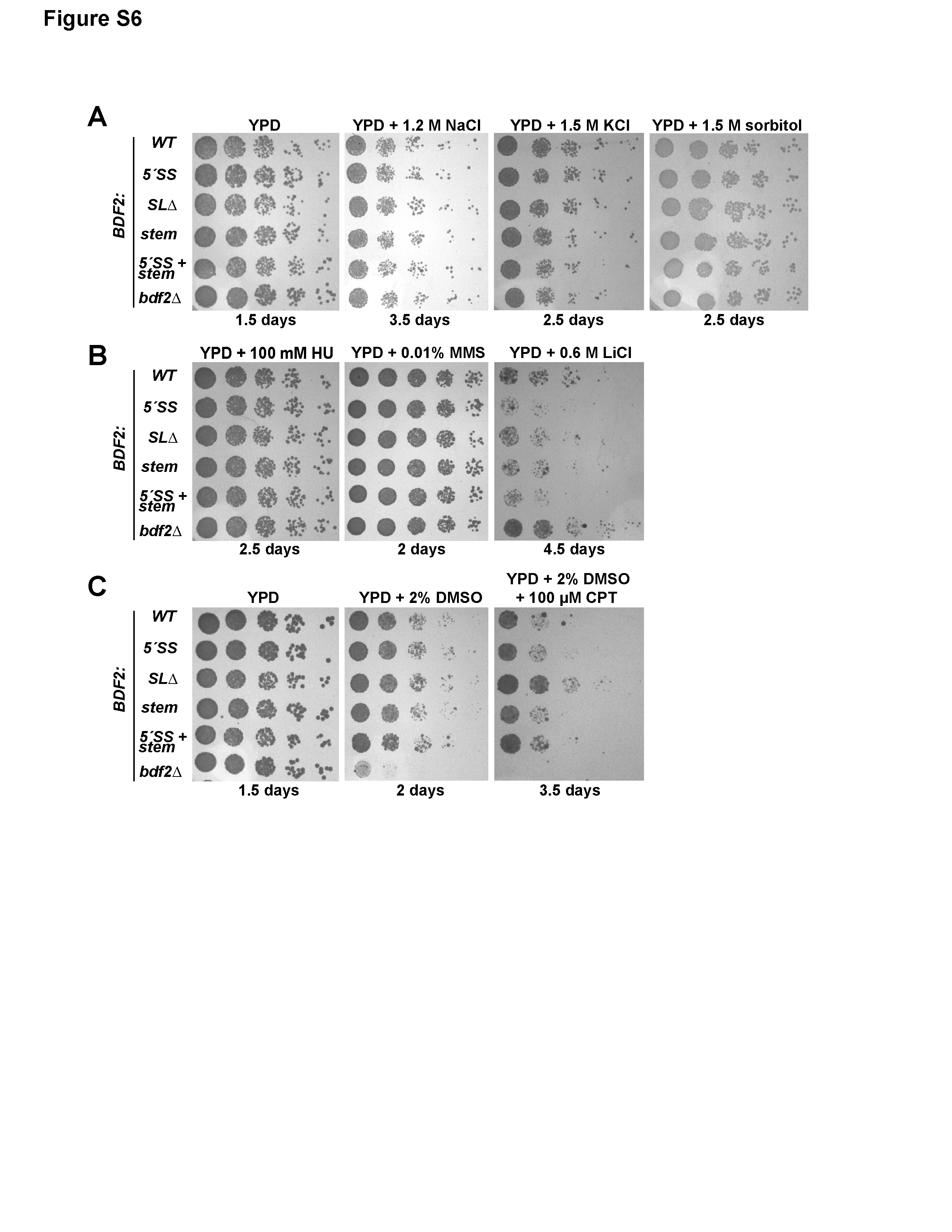

Supplement: Figure S6 — The wild-type strain and the specified BDF2 mutants were grown on plates with the indicated media at 30°C for the indicated number of days. (A) Inactivation of RMD or SMD of BDF2 mRNA has no effect on the growth of the wild-type background in high salt or hyperosmotic stress conditions. (B) Inactivation of RMD or SMD of BDF2 mRNA confers sensitivity to 0.6 M lithium chloride, but not methyl methanesulfonate (MMS) or hydroxyurea (HU). (C) Cells lacking BDF2 are hyper-sensitive to 2% dimethyl sulfoxide (DMSO), and do not grow on the combination of 2% DMSO and 100 µM camptothecin (CPT). (TIF) [file pgen.1004661.s006.tif]
